# In Silico and In Vitro Inhibition of SARS-CoV-2 PL^pro^ with Gramicidin D

**DOI:** 10.3390/ijms24031955

**Published:** 2023-01-19

**Authors:** Sara Protić, Nevena Kaličanin, Milan Sencanski, Olivera Prodanović, Jelena Milicevic, Vladimir Perovic, Slobodan Paessler, Radivoje Prodanović, Sanja Glisic

**Affiliations:** 1 University of Belgrade-Faculty of Chemistry, Studentski trg 12, 11000 Belgrade, Serbia; 2 University of Belgrade-Institute of Chemistry, Technology and Metallurgy, Njegoševa 12, 11000 Belgrade, Serbia; 3 Laboratory of Bioinformatics and Computational Chemistry, Institute of Nuclear Sciences Vinca, National Institute of the Republic of Serbia, University of Belgrade, 11001 Belgrade, Serbia; 4 University of Belgrade-Institute for Multidisciplinary Studies, Kneza Višeslava 1, 11030 Belgrade, Serbia; 5 Department of Pathology, University of Texas Medical Branch, Galveston, TX 77555, USA; 6 Institute for Human Infections and Immunity, University of Texas Medical Branch, Galveston, TX 77555, USA

**Keywords:** anti SARS-CoV-2, PL^pro^, COVID-19, gramicidin D, PL^pro^ candidate inhibitor

## Abstract

Finding an effective drug to prevent or treat COVID-19 is of utmost importance in tcurrent pandemic. Since developing a new treatment takes a significant amount of time, drug repurposing can be an effective option for achieving a rapid response. This study used a combined in silico virtual screening protocol for candidate SARS-CoV-2 PL^pro^ inhibitors. The Drugbank database was searched first, using the Informational Spectrum Method for Small Molecules, followed by molecular docking. Gramicidin D was selected as a peptide drug, showing the best in silico interaction profile with PL^pro^. After the expression and purification of PL^pro^, gramicidin D was screened for protease inhibition in vitro and was found to be active against PL^pro^. The current study’s findings are significant because it is critical to identify COVID-19 therapies that are efficient, affordable, and have a favorable safety profile.

## 1. Introduction

Finding an effective drug to prevent or treat COVID-19 is of utmost importance in the current pandemic. Developing a new treatment takes a significant amount of time; hence, repurposing drugs may be an efficient option for achieving a rapid response.

The inhibition of the virus papain-like protease (PL^pro^), which is essential in the life cycle of coronaviruses, has been considered a possible way to treat COVID-19 patients infected with SARS-CoV-2 [1]. The PL^pro^ is required for viral polyprotein processing to generate a functional replicase complex and enable viral spread [2]. PL^pro^ is a monomer with a canonical catalytic triad of Cys111/His272/Asp286 at its active site [2,3]. PL^pro^ cleaves ubiquitin and ISG15 to avoid host antiviral immune responses [3]. SARS-CoV-2 has a positive-sense, single-strand RNA genome with at least ten open reading frames (ORFs). The biggest, ORF1ab, which encodes two large overlapping polyproteins, pp1a and pp1ab, is required for viral replication and transcription and is cleaved by proteases to produce 16 non-structural proteins (NSP). The 3–6 NSP3 encodes PL^pro^, which detects the LXGG tetra-peptide motif between viral proteins nsp1 and nsp2, and nsp2 and nsp3. It processes the replicase polyprotein 1a (pp1a) and replicase polyprotein 1ab (pp1ab) on the N-termini into nsp1, nsp2, and nsp3, essential for viral replication [4]. Small peptides, as substrate-based inhibitors of the SARS-CoV-2 PL^pro^ enzyme, may be capable of unraveling COVID-19 disease [5]. Several rationally designed short peptides have been developed as as substrate-based inhibitors of PL^pro^ of SARS family viruses, demonstrating high selectivity for PL^pro^ binding and impeding viral multiplication [6]. Recently, two tetra-peptides were identified as CoV-2 PL^pro^ inhibitors that suppress viral replication in vitro and in cell lines [7]. The small peptides, known as antimicrobial peptides (AMPs), widely exist in nature and are an essential part of the innate immune systems of different organisms [8]. AMPs are the host’s first line of defense against pathogens, with a wide range of antimicrobial activity against Gram-positive and Gram-negative bacteria, fungi, and viruses [9]. They have a reputation as promising candidates for repurposing as antiviral agents, some of which have been documented to inhibit SARS-CoV-2 [10]. Because AMPs are less likely to develop resistance, they are considered promising in the fight against infectious diseases [9,11]. However, only a few AMPs (out of over three thousand discovered) have been approved by the FDA, with gramicidins, polymyxins, and nisins being the best-studied [9,12]. Gramicidins were previously shown to display antiviral activity against HIV and herpes simplex virus types 1 and 2 [13,14]. Gramicidin S, an antibacterial peptide with potent antibacterial and fungicidal activity [15], has recently been shown to have anti-SARS-CoV-2 activity in vitro [16]. According to the results of another molecular docking study, gramicidin S may have direct antiviral activity against the SARS-CoV-2 virus by binding to the SARS-CoV-2 spike glycoprotein and SARS-CoV-2 PL^pro^ [17].

In this study, as an extension of our previous drug repurposing research, we identified gramicidin D using the same in silico approach as in our previous study searching for SARS-CoV-2 PL^pro^ inhibitors [18]. Gramicidin D was selected as the peptide drug with the best in silico interaction profile with PL^pro^. In further experimental testing, it was found to be active against PL^pro^ in vitro.

## 2. Results

To identify potential PL^pro^ inhibitor candidates, we employed a virtual screening protocol with combined sequential filters based on long-range and short-range interactions, using the same approach as in our previous study [18]. Small molecule–protein interactions were examined using the informational spectrum method (ISM) for small molecules (ISM-SM). To discover potential PL^pro^ inhibitors among approved peptide drug candidates, we further searched Drugbank [19] using the ISM SM method [19] for approved drug candidates, using the frequencies characteristic of PL^pro^ F(0.383) and F(0.279) that we proposed in our previous study. As a result of this analysis, gramicidin D was selected as the best PL^pro^ peptide drug candidate inhibitor.

### 2.1. Molecular Docking

**Figure 1:**
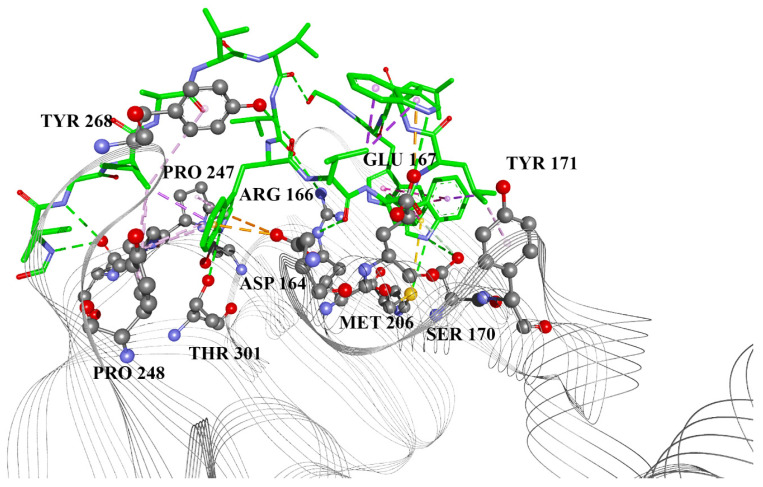
Gramicidin D docked into the catalytic site of PL^pro^. Green lines: hydrogen bonds; orange: electrostatic (including Pi-anion); yellow: Pi-sulfur; magenta: Pi-alkyl.

Since the activity of gramicidin S against SARS-CoV-2 was reported in addition to its in silico activity against PL^pro^ we compared the binding affinity of these two peptides by molecular docking. We carried out the molecular docking of gramicidin D and gramicidin S into the site of reported co-crystallized PL^pro^ inhibitors, using the PL^pro^–GRL 0617 complex structure (PDB ID 7CJM) [20]. The calculated binding energy from the docking poses for both molecules is −6.9 kcal/mol (Ki = 8.65 μM). Gramicidin D shows the presence of anion–π interaction with Asp 164, which is an important residue in terms of catalytic efficiency. Compared with the inhibitor GRL 0617, it interacts with Pro 247 and Pro 248 (Figure 6, Table 2 in Ref [18]). Additionally, interaction with Glu 167 was reported in the cases of Epicriptine and Ergometrine [18]. The docked conformation of gramicidin D is presented in Figure 1.

**Figure 2:**
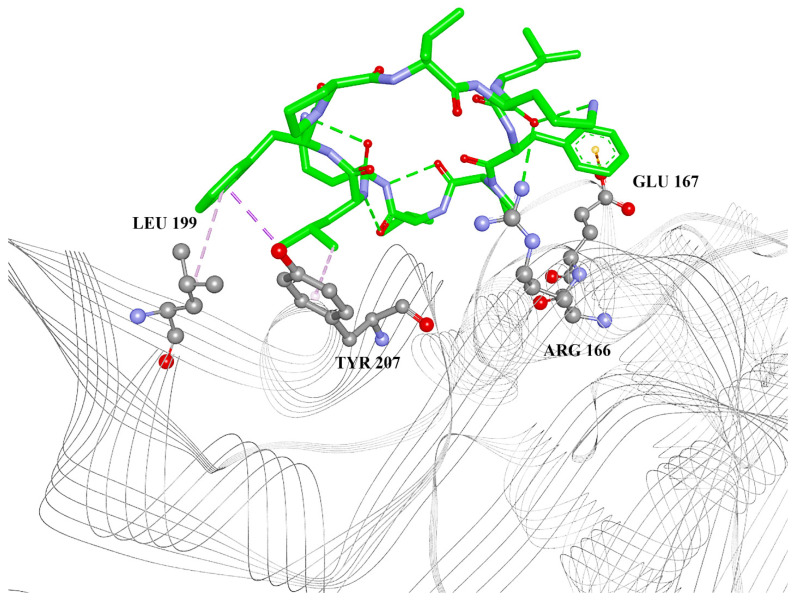
Gramicidin S docked into the catalytic site of PL^pro^. Green lines: hydrogen bonds; orange: electrostatic (including Pi-anion); yellow: Pi-sulfur; magenta: Pi-alkyl.

Gramicidin S shows interactions with Arg 166, Glu 167, Ser 170, and Leu 199 (Figure 2). Despite forming fewer intermolecular interactions with the PL^pro^ catalytic site compared with gramicidin D, the binding energies have the same value. Additionally, the contribution of hydrophobic interactions is greater in the case of gramicidin S than in the case of gramicidin D (Table 1). One would expect a stronger binding of gramicidin D to the catalytic site, but this is not the case. This is probably due to the cyclic constitution of gramicidin S and therefore less change in entropy during the binding process:

ΔG = ΔH − TΔS

As Gibbs free energy should have a negative value, in the binding process, by lowering the degrees of freedom of a ligand (causing a negative ΔS), a more drastic change is found in the case of a linearly flexible molecule than in the case of a cyclically flexible one. Therefore, the TΔS term would be more negative in the case of gramicidin D, producing a more positive contribution (−TΔS) to the binding energy.

The summary of protein–ligand intermolecular interactions for both gramicidin D and gramicidin S is presented in Table 1.

**Figure 3:**
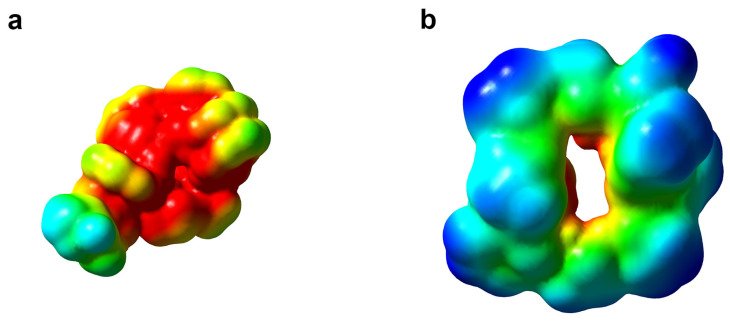
The ESP surfaces of gramicidin D (**a**) and gramicidin S (**b**). ESP potential is presented from a value of −20 (red) to −10 (blue).

As presented in Figure 3, there is a greater negative electrostatic potential present on the surface of gramicidin D than gramicidin S. However, the gramicidin S is a double-positively charged molecule. From the constitution of the surface amino acid residues of PL^pro^, it seems to be more hydrophobic than hydrophilic and more partially negatively charged. Therefore, one would expect gramicidin S to be a better binding ligand than gramicidin D. However, due to the complicated variation in electrostatic potential on the protein surface and the presence of negative and positive residues (Asp and Arg), the overall contact surface of gramicidin D is larger. Therefore, along with entropy effects, the difference in binding energy between two molecules is also due to the desolvation process, which should be higher for the charged species. Altogether, these effects manifest as protein–ligand contact surface interactions, solvation, and entropy effects.

### 2.2. Purification of PL^pro^

PL^pro^ was expressed in *Escherichia coli* BL21 STAR cells and purified to homogeneity by Ni-NTA affinity chromatography. The process of purification was analyzed by SDS electrophoresis on a 10% gel. The estimated molecular mass of the purified proteins is approximately 60 kDa, which is in accordance with the literature data for the weight of one GST-tagged PL^pro^ subunit, around 62 kDa.

### 2.3. PL^pro^ Inhibition with Gramicidin D

**Figure 4:**
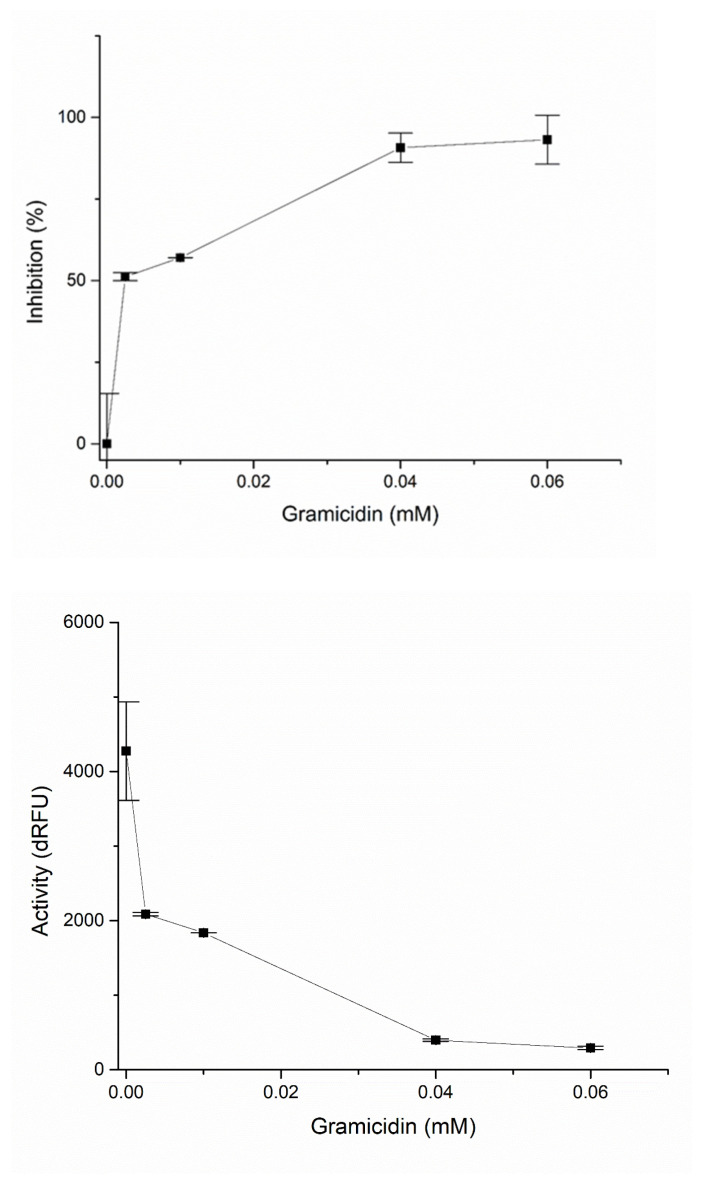
Inhibition (%) and activity (%) of PL^pro^ with different concentrations of gramicidin D.

The results of the present study show that gramicidin D has inhibitory activity against PL^pro^. With the addition of gramicidin D at 0.0025 mM, inhibition was around 50%, while increasing the concentration to 0.06 mM inhibited 93% of the original proteolytic activity of the PL^pro^ protease of the SARS-CoV-2 virus (Table 2, Figure 4).

Drug resistance is an important issue in health care, affecting therapeutic outcomes and necessitating novel drug design approaches. In silico drug repurposing is a quick and secure method for combating COVID-19. The SARS-CoV-2 PL^pro^ is a desirable therapeutic target because it promotes viral replication and modifies the host immune system, inhibiting the host’s antiviral innate immunological response and promoting antiviral immunity [2].

In the current study, an in silico strategy for repurposing approved drugs is employed to fight COVID-19. This study is extended to in silico drug repurposing analysis, from which we have identified the peptide drug gramicidin D as a candidate SARS-CoV-2 PL^pro^ inhibitor using the same in silico approach as in our previous study [18]. We used a virtual screening protocol with combined sequential filters based on long-range and short-range interactions to select candidates for PL^pro^ inhibitors. We examined small molecule–protein interactions using ISM-SM. This virtual technique may swiftly scan vast molecular libraries with minimal data preparation, using only the protein sequence and drug candidate’s SMILES molecular annotation. First, the Drugbank database was searched in silico using ISM-SM, followed by molecular docking. We found that gramicidin S and D have similar binding affinity, which is similar to the average docking scores of the compounds that bind the PL^pro^ inhibitor binding site obtained from our previous in silico study, identified as the best PL^pro^ candidate inhibitors [18]. In further experimental testing, gramicidin D was found to be active against PL^pro^ in vitro. That was not surprising, given that the presence of amino acid building blocks is especially seen in drugs and drug candidates, whose molecular targets naturally bind to amino acids or peptide structures. This is particularly accurate for protease inhibitors in antiviral therapy [21]. Gramicidin belongs to class the of AMPs, the host’s first line of defense against infections, with broad antimicrobial activity [9] and promise in the fight against infectious diseases because they are less susceptible to resistance [11]. Gramicidin D is among the few AMPs approved by the FDA, out of over three thousand discovered [9,12]. Gramicidin D is a well-tolerated antibiotic, but its application is limited to topical application due to its hemolytic side effect [22]. It is applied locally to treat infected wounds as well as eye, nose, and throat infections [23].

During the early stage of COVID-19, nasal multiciliated epithelial cells in the upper airway were discovered to be the primary target for SARS-CoV-2 infection and replication, implying that targeting these cells could be an excellent strategy for preventing SARS-CoV-2 transmission [24]. As a possible therapeutic treatment option, targeted intracellular drug delivery via nasal spray was proposed [24,25,26]. The nasal route is a desirable route for peptide drug delivery due to its simplicity of administration, adequate blood supply, and absorptive epithelium [27]. Moreover, various nasal sprays to combat SARS-CoV-2 have been proposed [28]. For that reason, the application of gramicidin D as a topical nasal treatment may potentially inhibit replication in the nasal epithelium, reducing the spread of the virus.

## 3. Materials and Methods

### 3.1. Data Preparation

The FASTA SARS-CoV-2 PL^pro^ sequence was downloaded from UNIPROT, and the corresponding IS was calculated. A set of 1490 approved drugs from Drugbank [19] with corresponding SMILES was subjected to IS and CS calculation with PL^pro^. All calculations were carried out using our in-house software. The PDB structure of PL^pro^ in complex with the inhibitor encoded by 7CJM [20] (GRL0167) was downloaded from the RCSB Protein Bank Database.

### 3.2. ISM

As a virtual spectroscopic method, the ISM has been successfully utilized to investigate the structure and function of diverse protein and DNA sequences. A thorough explanation of the ISM-based sequence analysis can be found elsewhere [29]. This method converts a sequence (protein or DNA) into a signal by giving each component (amino acid or nucleotide) a numerical value. These values represent the electron–ion interaction potential (EIIP) [30], which determines the electronic properties of amino acid/nucleotides and is essential for their intermolecular interactions. The following formulas can be used to calculate the EIIP descriptors:

Z* = ∑mi = 1niZi/N, (1)

EIIP = 0.25Z*sin(1.04πZ*)/2π,(2)

where i is the type of chemical element, Z is the valence of the i-th chemical element, n is the number of i-th chemical element atoms in the compound, m is the number of types of chemical elements in the compound, and N is the total number of atoms.

The EIIP signal is then transformed using the fast Fourier transform (FFT) into an information spectrum (IS) as a representation of a sequence in the form of a series of frequencies and amplitudes:

X(n) = ∑Nm = 1x(m)e − iπnmN, n = 1, 2, …, N/2,(3)

The summation index is denoted by “m”, where x(m) is the m-th member of a given numerical “signal” series (from a transformed, encoded primary protein sequence in our case), N is the total number of points in this series, n is the value of the discrete frequency (ranging from 1 on up to N/2) in the DFT, X(n) represents the discrete Fourier transformation amplitude coefficients corresponding to each discrete frequency n, and 2π × (n/N) is the phase angle at each given m in the amino acid series of the protein in question.

The virtual spectroscopic method enables the functional analysis of protein sequences without prior experimental data. ISM-SM, its extension for small molecules, was recently developed and published [31]. A small molecule is imported in SMILES notation and decoded by atomic groups into an array of corresponding EIIP values. Using FFT, the corresponding IS of a small molecule is computed. This spectrum is further multiplied by the IS of the protein receptor to obtain a cross-spectrum (CS). The cross-spectral function determines the common frequency characteristics of two signals. For discrete series, it is defined as follows:

S(n) = X(n) × Y(n)*, n = 1, 2, …, N/2,(4)

where X(n) and the DFT coefficients of the series x(m) and Y(n)* are complex conjugated DFT coefficients of the series Y(m).

It is possible to identify whether a protein interacts with small molecules and the corresponding binding site in the protein using common frequencies in CS.

### 3.3. Molecular Docking

The molecular docking of selected candidates into the crystal structure of PL^pro^ was carried out. The PDB structure of PL^pro^ complexed with the GRL0167 inhibitor (ID: 7CJM) [20] was downloaded from the RCSB Protein Bank database.

All ligands, waters, and ions were removed from the PDB file. Two grid boxes with dimensions of 24 × 24 × 24 Å were set to span all amino acid residues interacting with the co-crystallized inhibitor GRL 0617. The (x, y, z) center of the grid boxes was (26.0, 70.0, −1.0). Selected drugs from the previous step were converted from SMILES to 3D SDF and then to PDB files and protonated at physiological pH. Geometry optimization was carried in MOPAC 2016 [32] at the PM7 [33] level of theory. Default software settings for hydrophobic and hydrophilic terms in the docking search function were used. Exhaustiveness was set to 50. Molecular docking was carried out in Autodock Vina 1.1.2 [34]. Figures were made in BIOVIA Discovery Studio 2017, Schrodinger Maestro 11.1 and Origin 9.0 software.

### 3.4. Electrostatic Potential (ESP) Surface Calculations

ESP surfaces were calculated using the PM6 semiempirical method in Gaussian 09.D.01 [35].

### 3.5. Equipment

The thermostat “Environmental Shaker-Incubator ES-20” and the shaker “Thermo shaker TS-100 Biosan” were used to meet the needs of the growing microorganisms. The “Consort E122” system was used for protein electrophoresis. The HPLC AKTA system was used for enzyme purification. The TEKAN Infinite 200 Pro M Nano+ device was used to measure the enzyme activity by fluorescence.

### 3.6. Chemicals

Kanamycin, an antibiotic, was ordered from Invitrogen, California. Agar, peptone, and tryptone, components for medium preparation, were ordered from Torlak, Serbia. Other substances were ordered from Centrohem, Serbia.

### 3.7. Gene for PL^pro^

The gene for PL^pro^ ordered from Addgene was previously cloned into the pETM33 vector with N-terminal GST and His-tag, using NcoI and EcoRI restriction enzymes. Himera: His-GST-HRV_3C-PLP has 1677 bp with a MW of 63.9 kDa. The recommended expression conditions when using E. coli BL21 DE3 gold cells are growth at 37 °C, induction with IPTG at a final concentration of 1 mM, and expression for 16 h at 18 °C. The E. coli STAR strain was used for intracellular expression. Zinc acetate was added to the expression medium at a final concentration of 0.5 mM. The DH5α strain was used for the storage and propagation of plasmids.

### 3.8. Amino Acid Sequence of PL^pro^

DGEVRTIKVFTTVDNINLHTQVVDMSMTYGQQFGPTYLDGADVTKIKPHNSHEGKTFYVLPNDDTLRVEAFEYYHTTDPSFLGRYMSALNHTKKWKYPQVNGLTSIKWADNNCYLATALLTLQQIELKFNPPALQDAYYRARAGEAANFCALILAYCNKTVGELGDVRETMSYLFQHANLDSCKRVLNVVCKTCGQQQTTLKGVEAVMYMGTLSYEQFKKGVQIPCTCGKQATKYLVQQESPFVMMSAPPAQYELKHGTFTCASEYTGNYQCGHYKHITSKETLYCIDGALLTKSSEYKGPITDVFYKENSYTTT

### 3.9. Gramicidin D and S IUPAC Entry

The IUPAC entry for Gramicidin D and S was extracted from the Pubchem database.

Gramicidin D

For-Val-Gly-D-Leu-Ala-D-Val-Val-D-Val-Trp-D-Leu-Trp-D-Leu-Trp-D-Leu-Trp-Gly-ol

Available from: https://pubchem.ncbi.nlm.nih.gov/compound/Gramicidin-D, accessed on 12 January 2023 [36].

Gramicidin S

cyclo[Leu-D-Phe-Pro-Val-Orn-Leu-D-Phe-Pro-Val-Orn]

Available from: https://pubchem.ncbi.nlm.nih.gov/compound/Gramicidin-S [37].

### 3.10. Isolation and Purification

#### 3.10.1. Cell Lysis

After the protein expression, the collected cells were resuspended in lysis buffer (50 mM Na-phosphate buffer with 300 mM NaCl and 10 mM imidazole, pH 7.5). The sample was sonicated on ice with an ultrasound probe 10 times for 10 s, with a 20-s pause in between. After lysis, the cells were centrifuged for 20 min at 13,000 rpm. The supernatant was passed through a sterile 0.22 µL filter.

#### 3.10.2. Purification

PL^pro^ was purified by an HPLC system on a 5 mL Ni-NTA FF Sepharose column. For column equilibration, a 50 mM Na-phosphate buffer with 300 mM NaCl and 10 mM imidazole, pH 7.5, was used, while the same buffer with a gradient from 10 mM to 350 mM imidazole was used for protein elution. The change at 280 nm was monitored. Fractions of the purified protein were checked by SDS-PAGE electrophoresis (Supplementary Figure S1). Fractions containing the pure PL^pro^ (fractions 22–28) were pulled and dialyzed against a 50 mM Tris-HCl buffer, pH 7.5, with 150 mM NaCl and 1 mM DTT (Supplementary Figure S2). The isolated enzyme was stored in 10% glycerol at −20 °C.

#### 3.10.3. PL^pro^ Assay

The change in fluorescence was monitored for 35 min every 5 min, with excitation at 485 nm and emission at 535 nm. The total volume of the reaction mixture was 200 µL. The buffer in which the reaction took place was 20 mM Tris with 150 mM NaCl and 1 mM DTT at pH 7.5. The enzyme (10 µL) and 0.3 µL of the fluorescent substrate (Recombinant Human Ubiquitin Rhodamine 110 Protein) dissolved in DMSO were added to the reaction mixture, so that the final substrate concentration was 0.375 µM. Gramicidin D (2.5 μM, 10 μM, 40 μM, and 60 μM), dissolved in DMSO, was used as a possible inhibitor. Buffer was used as a blank, and buffer with the substrate was used as a control.

## 4. Conclusions

Drug resistance is a critical issue that has an impact on therapeutic outcomes and necessitates novel drug design approaches. In this paper, the potential antiviral effect of gramicidin D in vitro is shown. The findings of this study are significant because the development of COVID-19 treatments that are effective, affordable, and have a positive safety profile is crucial to lowering the burden of infectious diseases on the healthcare system.

## Figures and Tables

**Table 1:** Intermolecular interactions between SARS-CoV-2 PL^pro^ and gramicidin D/gramicidin S.

Amino Acid Residue	Interaction Type	Gramicidin D	Gramicidin S
ASP164	Pi-anion	Yes	No
ARG166	Conventional hydrogen bond	Yes	Yes
GLU167	Pi-anion	Yes	Yes
SER170	Conventional hydrogen bond	Yes	No
TYR171	Pi-alkyl	Yes	No
LEU199	Pi-alkyl	No	Yes
MET206	Conventional hydrogen bond, Pi-sulfur	Yes	No
TYR207	Pi-alkyl	No	Yes
PRO247	Pi-alkyl	Yes	No
PRO248	Conventional hydrogen bond, Pi-alkyl	Yes	No
THR301	Hydrogen bond	Yes	No

**Table 2:** Kinetic data for the inhibition of PL^Pro^ by gramicidin D.

	dRFU	Stdev	Stdev%	%Rezid.	%Inhibition
0	4274.67	660.27	15.45	100	0
0.0025	2087	25.46	1.22	48.82	51.18
0.01	1837	1.41	0.077	42.97	57.03
0.04	397.5	17.68	4.45	9.30	90.7
0.06	292.5	21.92	7.49	6.84	93.16

## Data Availability

Not applicable.

## Supplementary Materials

The following supporting information can be downloaded at: https://www.mdpi.com/article/10.3390/ijms24031955/s1.
