# Supplementary material for: In Silico and In Vitro Inhibition of SARS-CoV-2 PLpro with Gramicidin D
Source: Int J Mol Sci. 2023 Jan 19;24(3):1955. doi: 10.3390/ijms24031955 (PMC9915632; doi:10.3390/ijms24031955)
Supplement: Supplementary file 1 [file ijms-24-01955-s001.zip › Suppl Figure Captions.pdf]

Suppl. Figure Captions

Figure S1. Polyacrylamide gel electrophoresis (SDS-PAGE) on 10% separating gel of purified papain-like protease (F20-F25); (MM) molecular weight markers (14.4-116 kDa); fractions of purified papain-like protease (F27-F32).

Figure S2. The chromatogram
