# Supplementary figures and images for: In Silico and In Vitro Inhibition of SARS-CoV-2 PLpro with Gramicidin D
Source: Int J Mol Sci. 2023 Jan 19;24(3):1955. doi: 10.3390/ijms24031955 (PMC9915632; doi:10.3390/ijms24031955)

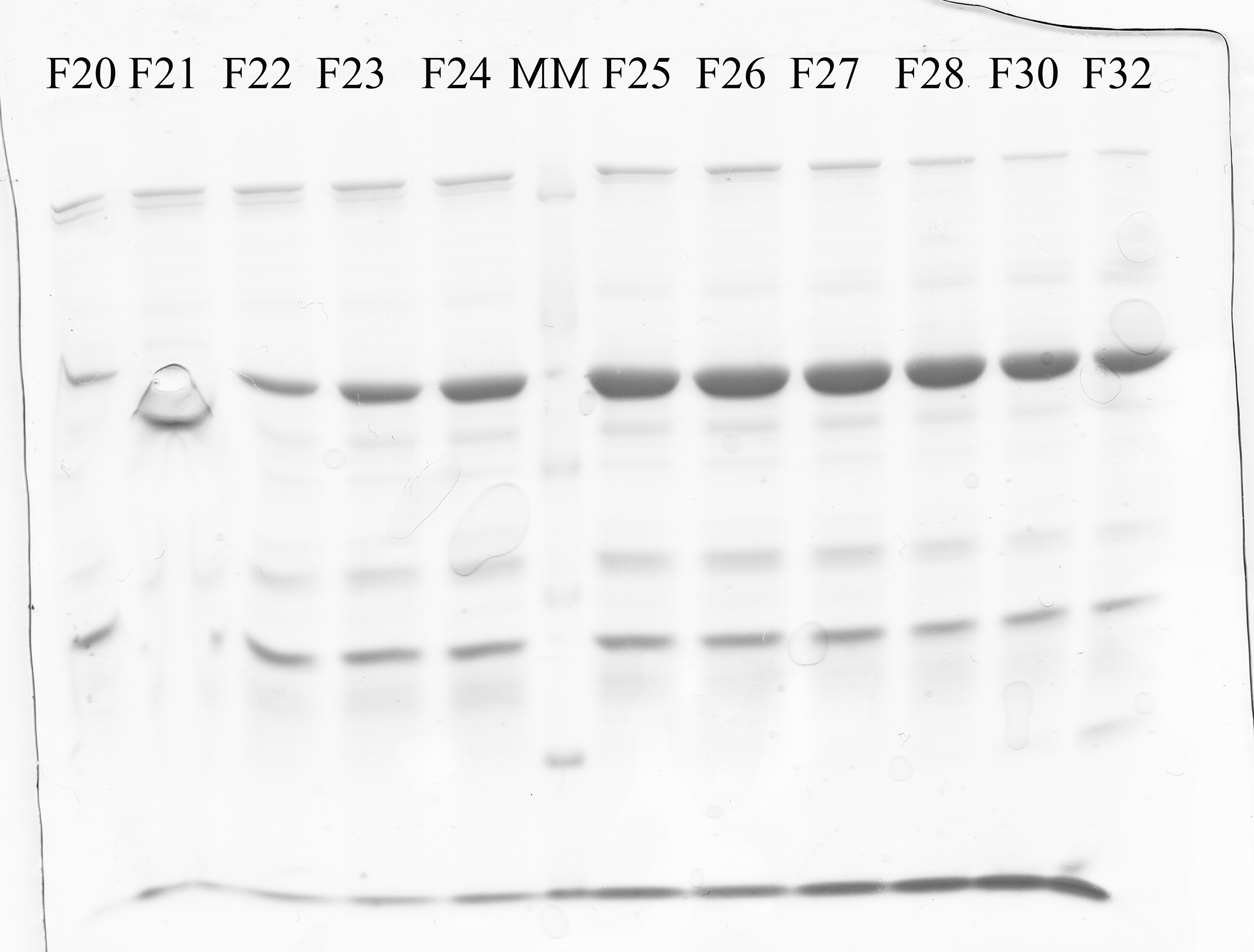

Supplement: Supplementary file 1 [file ijms-24-01955-s001.zip › Suppl. Figure 1.tif]
